# In Silico Exploration of Natural Antioxidants for Sepsis Drug Discovery

**DOI:** 10.3390/molecules30112288

**Published:** 2025-05-23

**Authors:** Celia María Curieses Andrés, Elena Bustamante Munguira, Celia Andrés Juan, Fernando Lobo, Eduardo Pérez-Lebeña, José Manuel Pérez de la Lastra

**Affiliations:** 1 Hospital Clínico Universitario of Valladolid, Avenida de Ramón y Cajal, 3, 47003 Valladolid, Spain; cmcuriesesa@saludcastillayleon.es (C.M.C.A.); ebustamante@saludcastillayleon.es (E.B.M.); 2 Cinquima Institute and Department of Organic Chemistry, Faculty of Sciences, Valladolid University, Paseo de Belén, 7, 47011 Valladolid, Spain; 3 Institute of Natural Products and Agrobiology (IPNA-CSIC), CSIC-Spanish Research Council, Avda. Astrofísico Francisco Sánchez, 3, 38206 San Cristóbal de la Laguna, Tenerife, Spain; fernando.lobo@csic.es; 4 Valladolid University Foundation, Paseo de Belén, 11, 47011 Valladolid, Spain; info@glize.eu

**Keywords:** molecular docking, antioxidants, sepsis, normal mode analysis, pathogenesis

## Abstract

Sepsis, a life-threatening condition characterized by immune dysregulation and organ damage, remains a significant clinical challenge. Natural antioxidant compounds (NAOs) such as quercetin, EGCG, resveratrol, curcumin, and chlorogenic acid have shown promising anti-inflammatory and anti-apoptotic effects in preclinical models of sepsis and related conditions, yet the molecular mechanisms underlying their actions remain incompletely defined. In this study, we performed comprehensive molecular docking analyses to investigate the binding affinities and interaction profiles of these NAOs with three key proteins central to inflammatory and apoptotic signaling: Toll-like receptor 4 (TLR-4), interleukin-1 receptor-associated kinase 1 (IRAK1), and caspase-3. Our results demonstrate that all five compounds exhibit favorable binding affinities with these targets, forming multiple hydrogen bonds and hydrophobic interactions with critical active site residues. Notably, curcumin and EGCG consistently displayed the strongest binding affinities across the three proteins, with docking scores comparable to or surpassing those of reference inhibitors. Resveratrol demonstrated highly stable binding poses, particularly with caspase-3, while quercetin and chlorogenic acid showed moderate but reproducible affinities. Overall, this study provides new mechanistic insights into how NAOs may target central mediators of inflammation and cell death. Experimental validation is essential to confirm these interactions, assess binding affinities, and fully elucidate the therapeutic potential of NAOs in sepsis.

## 1. Introduction

Sepsis is a life-threatening condition caused by a dysregulated host response to infection, leading to multi-organ dysfunction and high mortality rates [1]. Despite advances in critical care, sepsis remains a major global health challenge due to its complex pathophysiology [2]. The condition involves intricate interactions between innate immunity, inflammation, oxidative stress, and apoptosis, all of which contribute to the progression of organ failure [3]. Understanding these molecular mechanisms is essential for identifying novel therapeutic strategies to improve patient outcomes [4].

The innate immune system plays a crucial role in sepsis as the first line of defense against invading pathogens [5]. Toll-like receptor 4 (TLR4), a key pattern recognition receptor (PRR), is particularly significant in this context [6]. TLR4 recognizes lipopolysaccharide (LPS) from Gram-negative bacteria and triggers an inflammatory signaling cascade [7,8]. Upon activation, TLR4 recruits myeloid differentiation primary response 88 (MyD88), triggering interleukin-1 receptor-associated kinase 1 (IRAK1) phosphorylation and the subsequent activation of nuclear factor-κB (NF-κB) and mitogen-activated protein kinase (MAPK) pathways and leading to the production of pro-inflammatory cytokines such as tumor necrosis factor-α (TNF-α) and interleukin-6 (IL-6) [9,10,11,12]. While this response is critical for pathogen clearance, excessive activation of TLR4 contributes to the hyperinflammatory state observed in sepsis, exacerbating tissue damage and organ dysfunction [13,14].

A hallmark of sepsis is the dysregulated inflammatory response, often referred to as a “cytokine storm” [15]. This phenomenon involves both pro-inflammatory and anti-inflammatory processes occurring simultaneously, which can overwhelm the body’s ability to maintain homeostasis [16]. IRAK1 deficiency has been shown to attenuate early-phase cytokine responses in polymicrobial sepsis models, significantly improving survival rates (35% vs. 85% mortality in wild-type mice). However, redundant signaling pathways may compensate for IRAK1 absence at later stages, underscoring the importance of early intervention in sepsis management [10].

The interplay between oxidative stress and apoptosis further complicates sepsis progression [17]. Apoptosis, or programmed cell death, is significantly dysregulated in sepsis and contributes to immune suppression and organ failure [18,19]. Caspase-3, a central executioner caspase in apoptotic pathways, is activated via both intrinsic (mitochondrial) and extrinsic signals during sepsis [18,20]. Excessive apoptosis primarily affects lymphocytes and epithelial cells in vital organs such as the lungs and intestines [21]. This depletion of immune cells weakens the host’s ability to fight infections, increasing susceptibility to secondary infections and worsening clinical outcomes [22,23].

Given the multifaceted nature of sepsis pathophysiology, there is growing interest in exploring natural compounds with pleiotropic effects as potential therapeutic agents [24,25,26]. Natural products have long been recognized for their unmatched chemical diversity, evolutionary optimization for biological interactions, multi-target effects, and favorable safety profiles [27]. Many natural antioxidants (NAOs) have been consumed by humans for centuries, suggesting lower risks of unexpected side effects compared to synthetic drugs [28,29].

Among NAOs, compounds such as curcumin (from turmeric), chlorogenic acid (found in coffee and fruits), epigallocatechin gallate (EGCG; the main catechin in green tea), resveratrol (from grapes and berries), and quercetin (abundant in fruits and vegetables) have emerged as promising candidates for sepsis therapy [30,31]. These compounds exhibit well-documented anti-inflammatory and antioxidant properties that directly address key pathological processes in sepsis [32]. For example, curcumin has been shown to inhibit NF-κB activation and reduce oxidative stress [33]; chlorogenic acid acts as a potent free radical scavenger [34]; EGCG has demonstrated neuroprotective effects [35]; resveratrol exhibits cardioprotective properties [36]; and quercetin has been found to mitigate oxidative damage [37]. Importantly, these compounds have undergone extensive safety evaluations in clinical trials, generally showing favorable toxicity profiles even at high doses [38].

Despite their therapeutic potential, challenges such as poor bioavailability limit the clinical application of some NAOs. However, ongoing research into novel delivery systems—such as nanoformulations—offers promising solutions to enhance their pharmacokinetic properties and therapeutic efficacy [39].

To accelerate the discovery of novel therapies for sepsis based on natural compounds, computational approaches such as molecular docking have become invaluable tools [40]. Molecular docking enables the virtual screening of bioactive compounds by simulating their interactions with specific protein targets [41]. This technique allows researchers to rapidly identify potential drug candidates by predicting their binding affinities and interaction modes with target receptors. It reduces reliance on costly early-stage experimental studies while providing valuable insights into the molecular basis of compound activity [41,42].

While molecular docking has been widely used in drug discovery for various diseases, its application to modulating oxidative stress and inflammation in sepsis remains underexplored. This study aims to address this gap by investigating the interactions between key receptor targets implicated in sepsis pathophysiology—namely TLR4 (innate immunity), IRAK-1 (inflammation), and caspase-3 (apoptosis)—and several NAOs ligands: curcumin, chlorogenic acid, EGCG, resveratrol, and quercetin. Their ability to target multiple nodes in sepsis pathophysiology positions these phytochemicals as promising adjuvants to conventional therapies, potentially mitigating the limitations of single-target approaches in this heterogeneous syndrome [43].

To provide a deeper understanding of receptor dynamics in their unbound states, normal mode analysis (NMA) was employed. NMA offers insights into protein flexibility and functional mechanisms that are critical for understanding how these receptors interact with ligands under physiological conditions [44,45].

By leveraging computational methods such as molecular docking coupled with NMA, this study seeks to elucidate the molecular interactions between NAOs and key targets involved in sepsis pathophysiology. The findings aim to provide valuable insights into potential therapeutic strategies that harness the pleiotropic effects of natural compounds for critical care conditions like sepsis. Docking simulations may also reveal novel multi-target inhibitors—for example, a single polyphenol capable of simultaneously blocking TLR4 dimerization and caspase-3 activation-addressing sepsis heterogeneity more effectively than single-pathway drugs. Ultimately, this approach may pave the way for developing safer and more effective treatments that address multiple aspects of this devastating condition.

## 2. Materials and Methods

### 2.1. Selection of Receptor for Docking Studies

**Figure 1:**
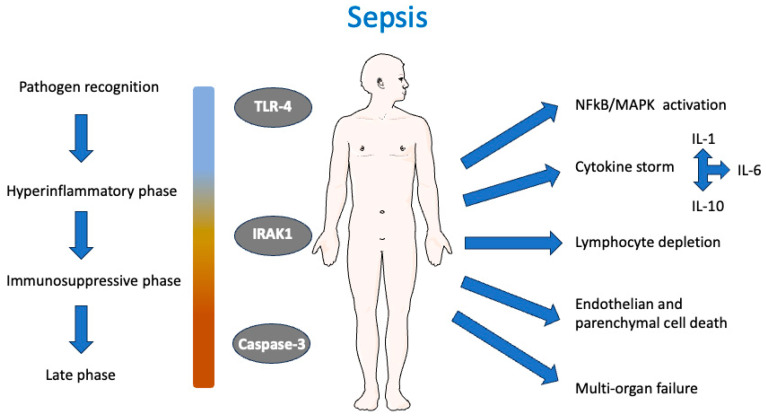
Schematic representation of the molecular and cellular events in sepsis and the role of TLR-4, IRAK1, and caspase-3. Sepsis is initiated via pathogen recognition through pattern recognition receptors, notably Toll-like receptor 4 (TLR-4), triggering a hyperinflammatory phase characterized by NFκB and MAPK activation and a subsequent cytokine storm involving pro-inflammatory (IL-1, IL-6) and anti-inflammatory (IL-10) cytokines. This is followed by an immunosuppressive phase and, in severe cases, progresses to a late phase with systemic consequences including lymphocyte depletion, endothelial and parenchymal cell death, and ultimately multi-organ failure. Interleukin-1 receptor-associated kinase 1 (IRAK1) and caspase-3 play central roles in mediating inflammatory signaling and apoptotic pathways, respectively.

The selection of TLR-4, IRAK1, and caspase-3 for docking studies in sepsis is grounded in their distinct yet interconnected roles across the syndrome’s pathobiological phases—pathogen recognition, hyperinflammation, and immunosuppression-induced organ dysfunction (Figure 1).

TLR-4 is the primary sensor for lipopolysaccharide (LPS) from Gram-negative bacteria, initiating innate immune responses via MyD88-dependent signaling. Targeting TLR-4 addresses the initial hyperinflammatory phase, where unchecked signaling amplifies tissue injury and multi-organ failure.

IRAK1 is a critical kinase downstream of TLR-4 and IL-1 receptor signaling, mediating NF-κB activation and cytokine release. The pharmacological inhibition of IRAK1 could temper the cytokine storm without fully ablating immune defenses, making it a strategic target for modulating early sepsis progression.

Caspase-3, the executioner protease of apoptosis, contributes to sepsis-induced lymphocyte depletion and immune paralysis, which impair pathogen clearance and increase secondary infection risk. Elevated caspase-3 activity in septic patients correlates with lymphocytopenia and early mortality. Inhibiting caspase-3 preserves immune cell populations (e.g., B cells, T cells) and reduces bacteremia in preclinical models, addressing the late immunosuppressive phase of sepsis. Furthermore, caspase-3 activation perpetuates organ dysfunction by promoting endothelial and parenchymal cell death

Protein structures for the selected receptors were retrieved from the UniProt database and the Protein Data Bank (PDB). Care was taken to ensure that high-resolution crystal structures or homology-modeled structures were used when available to enhance the accuracy of docking simulations. The protein structures for TLR4 (PDB ID: 3FXI), IRAK1 (PDB ID: 6BFN), and Caspase-3 (PDB ID: 6BDV) were obtained from the Protein Data Bank (PDB) database (https://www.rcsb.org/, accessed on 14 May 2025). ChimeraX [46] was used to preprocess the structure, removing solvent, ligands, and other protein chains. Details of the selected receptors are summarized in Table 1.

### 2.2. Selection of Ligands for Docking Studies

Five natural compounds were chosen: quercetin, epigallocatechin gallate (EGCG), resveratrol, curcumin, and chlorogenic acid. The selection of ligands for docking studies was guided by their well-documented natural antioxidant properties, their multifaceted biological activities, and favorable safety profiles demonstrated in clinical trials (Table 2). The three-dimensional structures of the selected ligands were obtained from the PubChem database in SDF and SMILES format.

### 2.3. Molecular Docking

Docking studies were performed using AutoDock 4.2.6 [47], with default parameters applied for both grid generation and docking simulations. Ligand and receptor structures were prepared using AutoDockTools 1.5.7, and grid maps were generated with AutoGrid, centering the grid on the corresponding binding pockets and using a box size of 40 × 40 × 40 Å. Each docking simulation was run 10 times using the Lamarckian Genetic Algorithm [48].

The binding free energy for each pose was estimated as the sum of van der Waals interactions, electrostatic forces, hydrogen bonding, desolvation effects, and torsional entropy contributions, according to the AutoDock scoring function [49].

As a metric of docking consistency, the root-mean-square deviation (RMSD) was calculated between the most stable pose and all other poses with binding energies within 1 kcal/mol of it.

To benchmark the docking results, active and inactive ligands were selected for both TLR4 and Caspase-3 from the ChEMBL database [50]. For TLR4, the active compound was CHEMBL5174883 [51], while the inactive one was Carvedilol [52]. For Caspase-3, the active ligand was CHEMBL456799 [53] and the inactive one was CHEMBL1242700 [54]. For IRAK1 we used the DL1 ligand (CHEMBL256713) from the PDB structure and performed a re-docking study to validate our approach.

## 3. Results and Discussion

Molecular docking has emerged as a powerful tool in drug discovery, enabling the rapid screening and prediction of interactions between therapeutic compounds and target proteins [55,56]. In our study, we applied this computational approach to explore the potential of five natural antioxidant compounds (NAOs) with established safety profiles (Table 2), for their ability to inhibit or modulate key enzymes and receptors involved in sepsis, including TLR-4, IRAK1, and caspase-3.

Given the multifaceted nature of sepsis, characterized by immune dysregulation, inflammation, oxidative stress, apoptosis, and coagulation abnormalities, our in silico strategy provides a promising avenue for identifying effective, low-toxicity interventions targeting this complex condition (Figure 1). Previous studies have demonstrated the utility of molecular docking for evaluating the antioxidant potential of natural compounds. For instance, Akash et al. (2022) explored myricetin derivatives to find a new bioactive molecule to treat breast and lung cancer using docking techniques to assess binding affinities and interactions with target proteins [57]. Similarly, Fisetin and other NAOs have been explored by computational methods against polymerase of Dengue virus [58] and for the treatment of Alzheimer’s disease [59]. These studies highlight the growing interest in leveraging computational tools to understand the therapeutic potential of NAOs.

### 3.1. Docking Validation Using Re-Docking

**Figure 2:**
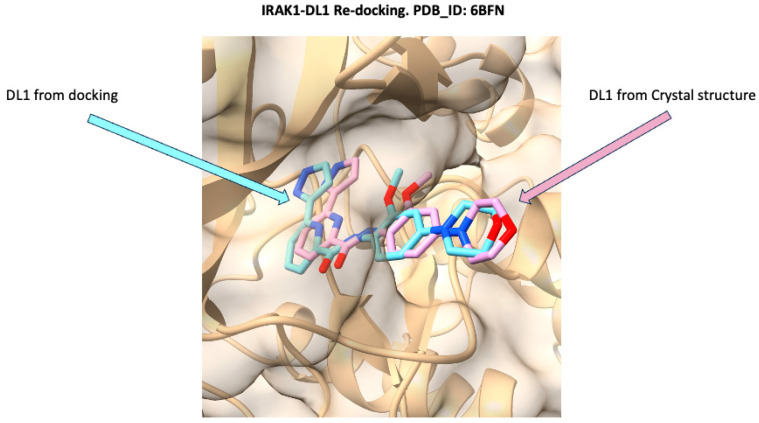
Superposition of the docked pose and crystallographic conformation of DL1 within the IRAK1 binding site (PDB ID: 6BFN). The DL1 ligand from the docking simulation is shown in cyan, while the DL1 ligand from the crystal structure is depicted in pink. The RMSD was 1.17 Å.

To validate the reliability of the docking protocol, a re-docking experiment was performed using the IRAK1-DL1 complex (PDB ID: 6BFN). As shown in Figure 2, the binding pose of DL1 obtained from the docking simulation (cyan) is closely superimposed with the DL1 conformation observed in the crystal structure (pink) within the IRAK1 binding site. This high degree of overlap, with an RMSD of 1.17 Å, demonstrates that the docking methodology accurately reproduces the experimentally determined binding mode of DL1. The successful re-docking supports the validity of the docking parameters [60] and provides confidence in the subsequent virtual screening and binding mode analyses conducted in this study.

### 3.2. Docking of NAOs with TLR4

**Figure 3:**
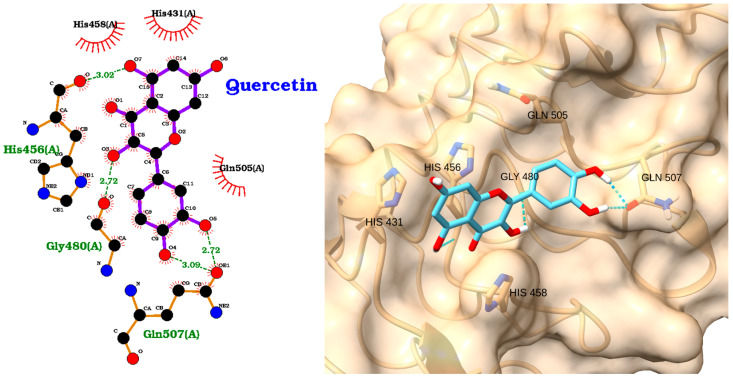
Two-dimensional interaction diagram (**left**) and three-dimensional binding pose (**right**) of quercetin docked to the TLR4 receptor. The 2D diagram highlights hydrogen bonds between quercetin and residues His456, Gly480, and Gln507 (green labels), as well as hydrophobic interactions with His431 and His458 (red arcs). The 3D structure shows quercetin (cyan) positioned within the TLR4 binding pocket, forming key interactions with the labeled residues.

**Figure 4:**
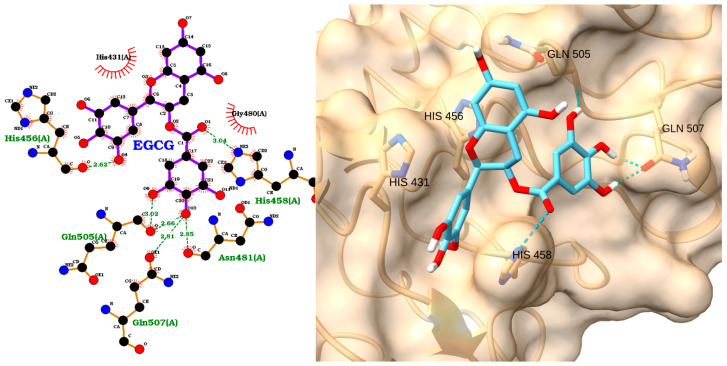
Two-dimensional interaction diagram (**left**) and three-dimensional binding pose (**right**) of EGCG docked to TLR4. EGCG forms multiple hydrogen bonds with His456, Gln505, Gln507, His458, and Asn481 and hydrophobic contacts with His431 and Gly480.

**Figure 5:**
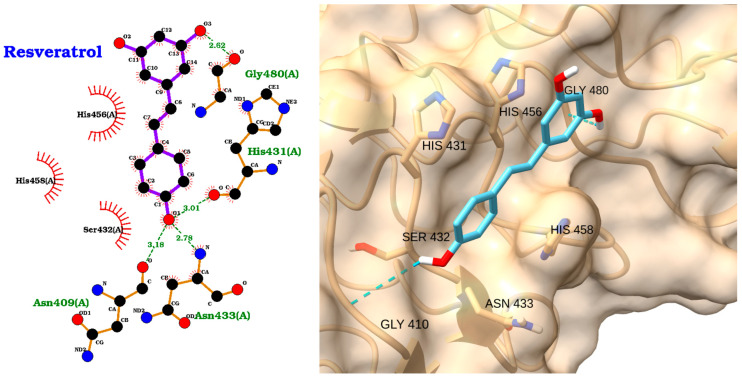
Two-dimensional interaction diagram (**left**) and three-dimensional binding pose (**right**) of resveratrol docked to the TLR4 receptor. The 2D diagram highlights hydrogen bonds between resveratrol and Gly480, His431, Ser432, Asn433, and Asn409, as well as hydrophobic interactions with His456 and His458. The 3D structure shows resveratrol (cyan) positioned within the TLR4 binding pocket, forming key contacts with the labeled residues.

**Figure 6:**
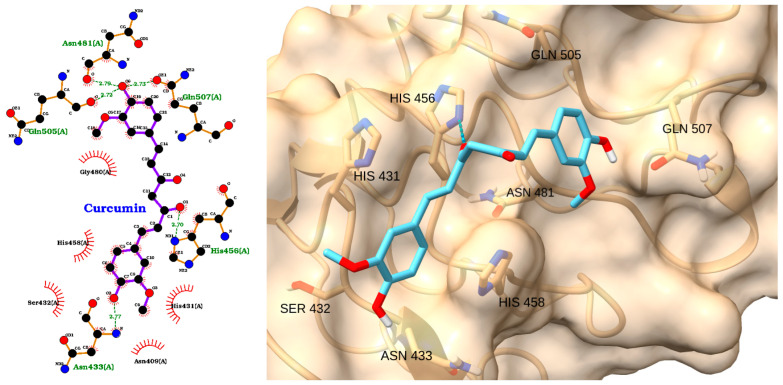
Two-dimensional interaction diagram (**left**) and three-dimensional binding pose (**right**) of curcumin docked to TLR4. Curcumin engages in hydrogen bonding with Gln505, Gln507, His456, and Asn433 and hydrophobic interactions with His431 and His458.

**Figure 7:**
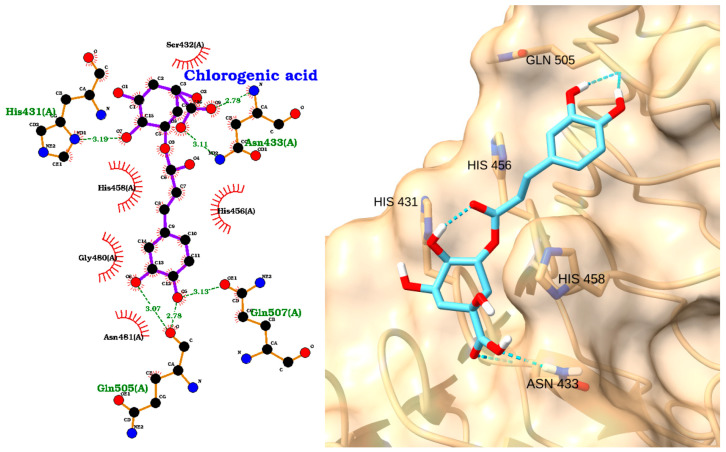
Two-dimensional interaction diagram (**left**) and three-dimensional binding pose (**right**) of chlorogenic acid docked to TLR4. Hydrogen bonds with His456, Gln505, Gln507, and Asn433 and hydrophobic contacts with His431 and His458 are depicted.

**Figure 8:**
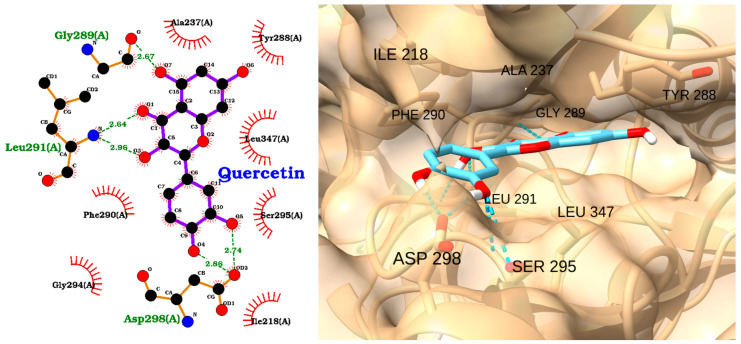
Two-dimensional interaction diagram (**left**) and three-dimensional binding pose (**right**) of quercetin docked to the IRAK1 active site. The 2D diagram illustrates hydrogen bonds (dashed green lines) between quercetin and residues Gly289, Leu291, Asp298, and Ser295, as well as hydrophobic interactions (red arcs) with Ala237, Tyr288, Leu347, and Phe290. The 3D structure shows quercetin (cyan) positioned within the IRAK1 binding pocket, highlighting its orientation and key interactions with surrounding residues.

Toll-like receptor 4 (TLR4) plays a pivotal role in the innate immune system and has emerged as a critical factor in sepsis. While Khan et al. identified anomalin, baicalein, and other compounds as TLR4 binders [61], the shared observation of ligand-receptor interactions supports the concept that targeting TLR4 can influence the inflammatory cascade. To explore potential binding sites of NAOs (NAOs) on TLR4, we performed local docking on the residues His-456 and His-458. According to the information provided in the UniProt entry of TLR-4 (O00206), these residues are responsible for inflammatory responses triggered via nickel (Ni^2+^). This same approach was previously reported for docking studies of TLR-4 using narciclasine [62]. The molecular docking analysis of NAOs against the TLR4 receptor revealed distinct binding affinities and interaction patterns, as illustrated in Figure 3, Figure 4, Figure 5, Figure 6, Figure 7 and Figure 8. The docking scores and RMSD values are summarized in Table 3, comprising compounds as positive and negative controls.

The docking study reveals quercetin as the top-performing ligand with the strongest binding affinity (score: −5.20 kcal/mol), though its relatively high RMSD (2.28 Å) suggests moderate pose stability.

The 2D interaction diagram (Figure 3, left) shows that quercetin forms multiple hydrogen bonds with key residues, including His456, Gly480, and Gln507, with bond distances ranging from 2.72 to 3.09 Å. Additional hydrophobic contacts with His431 and His458 further stabilize the ligand within the binding pocket. The 3D visualization (Figure 3, right) confirms the optimal positioning of quercetin, highlighting its extensive network of polar and hydrophobic interactions. Recent studies have shown that quercetin can inhibit TLR4 signaling in dendritic cells, which corroborates our findings [63].

The TLR4 docking with EGCG (Score: −5.15 kcal/mol) exhibits nearly equivalent binding strength but superior stability, as evidenced by its exceptionally low RMSD (0.36 Å), indicating a highly reproducible and stable interaction with the target (Table 3). The 2D interaction map (Figure 4, left) reveals hydrogen bonds with His456, Gln505, Gln507, His458, and Asn481, as well as hydrophobic interactions with His431 and Gly480. The 3D structure (Figure 4, right) shows EGCG deeply embedded in the TLR4 binding site, forming an extensive hydrogen-bonding network through its multiple hydroxyl groups. This complex binding mode is consistent with previous research demonstrating EGCG’s ability to downregulate TLR4 expression and inhibit its signaling pathway at physiologically relevant concentrations [64].

The docking analysis of resveratrol with the TLR4 receptor reveals a favorable binding mode, supported by a docking score of −4.60 kcal/mol and an RMSD of 2.12 Å (Table 3). The 2D interaction diagram (Figure 5 left) demonstrates that resveratrol forms multiple hydrogen bonds with key residues in the TLR4 binding pocket, including Gly480 (2.62 Å), His431 (2.91 Å), Ser432 (3.01 Å), Asn433 (2.78 Å), and Asn409 (3.18 Å). In addition, hydrophobic interactions are observed with His456 and His458, further stabilizing the ligand within the active site. The 3D visualization confirms that resveratrol is well accommodated in the binding pocket, with its aromatic rings oriented to maximize hydrophobic contacts and its hydroxyl groups participating in hydrogen bonding (Figure 5 right). These interactions suggest that resveratrol can effectively engage TLR4, potentially modulating receptor activity. The moderate binding affinity and stable pose, as indicated by the docking score and RMSD, support the potential of resveratrol as a TLR4 modulator in the context of sepsis.

Curcumin displayed a moderate binding affinity to TLR4 (score: −4.46 kcal/mol) and an RMSD of 1.46 Å (Table 3). As depicted in Figure 6 left, curcumin interacts with TLR4 via hydrogen bonds with Gln505, Gln507, His456, and Asn433 and hydrophobic contacts with His431 and His458. The 3D pose demonstrates curcumin’s linear structure, allowing it to span the binding pocket and engage several residues (Figure 6 right). Curcumin effectively blocks the TLR4/NF-κB signaling pathway, resulting in decreased cytokine expression and oxidative stress [65,66]. Importantly, experimental models of sepsis have demonstrated that curcumin can alleviate organ dysfunction, including damage to the brain, lungs, and kidneys, and improve overall survival rates.

Our finding aligns with previous studies that have shown curcumin’s ability to inhibit TLR4 signaling in non-small cell lung cancer cells [67].

Chlorogenic acid showed the weakest binding among the four antioxidants, with a docking score of −3.76 kcal/mol and an RMSD of 2.66 Å (Table 3). The 2D interaction diagram (Figure 7, left) indicates hydrogen bonds with His456, Gln505, Gln507, and Asn433 and hydrophobic contacts with His431 and His458. The 3D visualization (Figure 7, right) illustrates chlorogenic acid occupying a similar region within the TLR4 pocket, but with fewer stabilizing interactions compared to the other compounds. This extensive interaction network may explain chlorogenic acid’s reported ability to suppress TLR4 protein expression in spinal cord injury models [68].

The critical role of TLR-4 in the pathogenesis of sepsis [69] supports our investigation into NAOs as potential modulators of this receptor. The strong binding affinities observed, particularly for quercetin and EGCG, suggest that these compounds could effectively modulate TLR--4 signaling in conditions where TLR4 hyperactivation contributes to pathology. This modulation could potentially lead to reduced inflammation and improved outcomes. Despite these promising developments, challenges remain in translating TLR4-targeted therapies into clinical practice.

### 3.3. Docking of NAOs with IRAK1

IRAK1 is a critical mediator of TLR and interleukin-1 receptor (IL-1R) signaling, driving hyperinflammation in sepsis. Clinical and preclinical studies highlight IRAK1’s detrimental role: IRAK1-deficient mice exhibit reduced cytokine storms, attenuated neutrophil activation, and improved survival in polymicrobial sepsis [10], while a hyperactive IRAK1 haplotype in humans correlates with heightened NF-κB activation, organ failure, and mortality [70].

Targeting IRAK1 with inhibitors offers a promising therapeutic strategy. For instance, quinone-derived compounds (e.g., 1,4-naphthoquinone) selectively inhibit IRAK1 kinase activity, suppressing TLR4-mediated cytokine release and NF-κB signaling [71]. By blocking IRAK1, these compounds mitigate early-phase inflammatory responses without entirely abolishing host defense, preserving a balanced immune reaction [72]. NAOs like polyphenols or oligosaccharides may similarly modulate IRAK1 activity, reducing oxidative stress and inflammation while preventing immune paralysis. This dual action—curbing cytokine storms and apoptosis—addresses sepsis’s multifactorial pathology [73].

Curcumin exhibited the strongest predicted binding affinity among the tested natural compounds, with a docking score of −9.05 kcal/mol and an RMSD of 2.00 Å, closely approaching the performance of the positive control ligand (score: −9.30 kcal/mol; RMSD: 0.79 Å). EGCG also showed a high binding affinity (score: −8.86 kcal/mol), though with a slightly higher RMSD (1.85 Å), indicating a stable but somewhat more variable pose. Quercetin demonstrated a favorable binding score of −8.18 kcal/mol and an exceptionally low RMSD of 0.27 Å, suggesting both strong affinity and high pose reproducibility. Resveratrol (score: −6.72 kcal/mol; RMSD: 0.38 Å) and chlorogenic acid (score: −7.14 kcal/mol; RMSD: 2.13 Å) displayed moderate binding affinities, with resveratrol showing particularly good pose stability (Table 4).

The docking analysis of quercetin with IRAK1 reveals a strong and stable binding mode, as supported by a docking score of −8.18 kcal/mol and an exceptionally low RMSD of 0.27 Å (Table 4). The 2D interaction diagram (Figure 8, left) highlights multiple hydrogen bonds formed between quercetin and key amino acid residues within the IRAK1 binding site. Specifically, quercetin establishes hydrogen bonds with Gly289 (2.64 Å), Leu291 (2.98 Å), Asp298 (2.86 Å), and Ser295 (2.74 Å), indicating strong polar interactions that anchor the ligand within the active site. Additional hydrophobic contacts are observed with residues such as Ala237, Tyr288, Leu347, and Phe290, further stabilizing the complex (Figure 8 left).

The 3D visualization (Figure 8, right) confirms that quercetin is well accommodated within the IRAK1 binding pocket, with its aromatic rings and hydroxyl groups optimally oriented to maximize both hydrogen bonding and hydrophobic interactions. The close spatial proximity of quercetin to the key residues is consistent with the observed low RMSD (Table 4), reflecting a highly reproducible and reliable binding pose.

These results suggest that quercetin is a promising candidate for IRAK1 inhibition, with a binding affinity and pose stability comparable to those of established inhibitors. Its ability to form multiple stabilizing interactions within the IRAK1 active site supports its potential as a modulator of inflammatory signaling in sepsis. Recent studies have demonstrated that quercetin inhibits IL-1β-induced inflammation and cartilage degradation by suppressing the IRAK1/NLRP3 signaling pathway. In a rat osteoarthritis model, quercetin administration led to reduced expression of IRAK1, NLRP3, and caspase-3, along with decreased inflammation and apoptosis in both in vivo and in vitro settings. Rescue experiments further confirmed that the protective effects of quercetin on chondrocytes were mediated specifically through IRAK1/NLRP3 pathway inhibition [74,75]. Additionally, a 2024 study found that quercetin directly targets Syk/Src/IRAK1 to inhibit LPS-induced macrophage activation and cytokine storm, a process highly relevant to sepsis pathology. Quercetin significantly reduced the release of pro-inflammatory cytokines (IL-6, TNF-α, IL-1β) in LPS-activated macrophages, supporting its anti-inflammatory efficacy via IRAK1 modulation [76].

**Figure 9:**
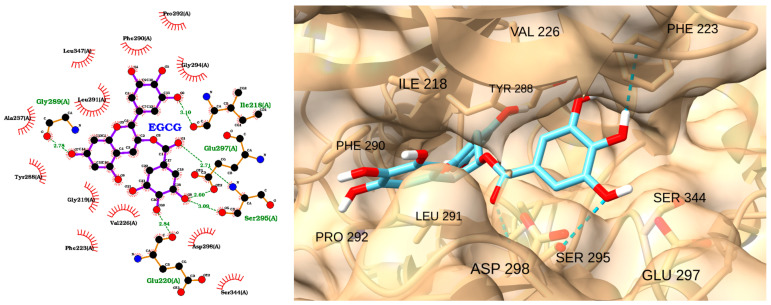
Two-dimensional interaction diagram (**left**) and three-dimensional binding pose (**right**) of EGCG docked to the IRAK1 active site. The 2D diagram shows hydrogen bonds (dashed green lines) between EGCG and residues Gly289, Glu297, Ser295, and Asp298, as well as hydrophobic contacts (red arcs) with Phe290, Leu291, Ile218, and Tyr288. The 3D structure displays EGCG (cyan) positioned within the IRAK1 binding pocket, forming key interactions with surrounding residues.

The docking analysis of EGCG with IRAK1 demonstrates a strong and favorable binding mode, as indicated by a docking score of −8.86 kcal/mol and an RMSD of 1.85 Å (Table 4). The 2D interaction diagram (Figure 9, left) reveals that EGCG forms multiple hydrogen bonds with key residues in the IRAK1 active site, including Gly289, Glu297, Ser295, and Asp298. The ligand is also stabilized by hydrophobic interactions with several residues such as Phe290, Leu291, Ile218, and Tyr288. These interactions suggest that EGCG is well accommodated within the binding pocket, engaging both polar and nonpolar contacts.

The 3D visualization (Figure 9, right) confirms the binding orientation of EGCG within the IRAK1 pocket. EGCG is shown forming hydrogen bonds (dashed green lines) with residues Ser295, Asp298, and Glu297, and it is surrounded with hydrophobic residues including Phe223, Val226, Ile218, Leu291, and Phe290. The aromatic rings and multiple hydroxyl groups of EGCG facilitate these extensive interactions, contributing to its high binding affinity and stable pose.

Molecular docking and modeling further revealed that EGCG binds to the ATP-binding site of IRAK1. In vitro kinase assays showed that EGCG inhibited IRAK1 activity at a 1 μM concentration by approximately 66%. These interactions stabilize EGCG within the kinase domain, resulting in reversible inhibition of IRAK1 activity [77]. Functionally, EGCG’s inhibition of IRAK1 leads to reduced activation of downstream inflammatory signaling pathways, including NF-κB and MAPKs, and it suppresses the production of pro-inflammatory cytokines such as IL-6 and IL-8 in human synovial fibroblasts. This effect was confirmed both in vitro and in a rat model of inflammatory arthritis, where EGCG administration ameliorated disease by dampening IRAK1-dependent signaling [77,78]. In summary, EGCG directly inhibits IRAK1 kinase activity and downstream inflammatory responses, supporting its potential as a natural IRAK1 inhibitor in inflammatory diseases and validating our docking results.

For resveratrol, while there is robust evidence for its anti-inflammatory properties and its ability to modulate key inflammatory signaling pathways (e.g., TLR4/MyD88/NF-κB), direct evidence for specific inhibition of IRAK1 is less well established. Most studies highlight resveratrol’s effects on upstream or parallel pathways, such as Sirt1 activation and TLR4/NF-κB suppression, rather than direct IRAK1 inhibition [79,80].

**Figure 10:**
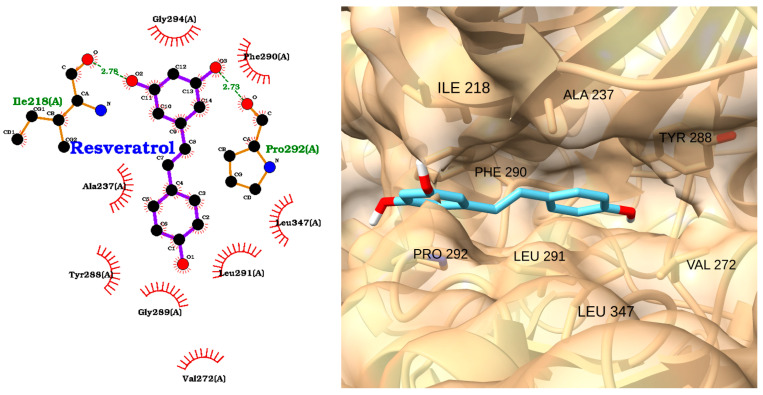
Two-dimensional interaction diagram (**left**) and three-dimensional binding pose (**right**) of resveratrol docked to the IRAK1 active site. In the 2D diagram, resveratrol forms hydrogen bonds with Ile218 and Pro292 (green labels, bond distances: 2.78 Å) and hydrophobic interactions (red arcs) with Ala237, Tyr288, Gly289, Phe290, Leu291, Val272, and Leu347. The 3D structure shows resveratrol (cyan) positioned within the IRAK1 binding pocket, highlighting its orientation and key contacts with surrounding residues.

The docking analysis of resveratrol with IRAK1 reveals a moderate binding affinity, as indicated by a docking score of −6.72 kcal/mol and an RMSD of 0.38 Å (Table 4). The 2D interaction diagram (Figure 10, left) shows that resveratrol forms hydrogen bonds with key residues Ile218 and Pro292, both with bond distances of 2.78 Å. These polar interactions help anchor resveratrol within the IRAK1 active site. Additionally, the ligand is stabilized by multiple hydrophobic contacts with residues such as Ala237, Tyr288, Gly289, Phe290, Leu291, Val272, and Leu347, as depicted by the red arcs in the diagram.

The 3D visualization (Figure 10, right) further confirms that resveratrol is well accommodated within the IRAK1 binding pocket. The ligand (shown in cyan) is oriented to maximize hydrophobic interactions along the binding groove, with its aromatic rings in close proximity to Phe290, Leu291, and Leu347. The low RMSD value reflects a highly stable and reproducible binding pose.

Overall, these results suggest that resveratrol could effectively interact with the IRAK1 active site through a combination of hydrogen bonding and hydrophobic contacts. While its binding affinity is moderate compared to other tested compounds, the stable pose and favorable interactions position resveratrol as a multi-target anti-inflammatory agent with the potential to modulate IRAK1 activity, offering a rational strategy to mitigate dysregulated immune responses in sepsis. Further mechanistic studies could refine its therapeutic application in IRAK1-driven inflammatory conditions.

Curcumin, a polyphenolic compound derived from *Curcuma longa*, has demonstrated significant anti-inflammatory and organ-protective effects in various preclinical models of sepsis and related disorders. Multiple studies have shown that curcumin administration reduces mortality, attenuates organ injury, and suppresses the production of pro-inflammatory cytokines in animal models of septic acute kidney injury and myocardial injury [81,82,83]. Mechanistically, curcumin’s beneficial effects are linked to the inhibition of key innate immune signaling pathways, including the downregulation of TLRs and their downstream adapters such as MyD88 and IRAK family kinases [84]. Specifically, curcumin and its analogs have been reported to reduce IRAK1 phosphorylation and expression, thereby disrupting the activation of NF-κB and MAPK pathways and limiting the inflammatory cascade.

**Figure 11:**
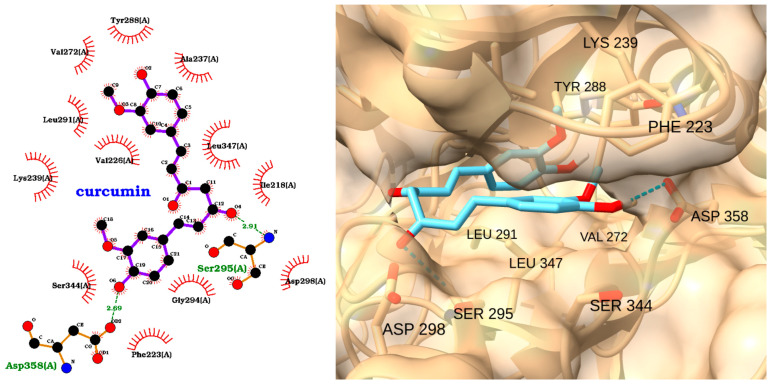
Two-dimensional interaction diagram (**left**) and three-dimensional binding pose (**right**) of curcumin docked to the IRAK1 active site. The 2D diagram highlights hydrogen bonds (dashed green lines) between curcumin and residues Ser295(A) and Asp358(A), as well as hydrophobic interactions (red arcs) with Tyr288(A), Val272(A), Leu291(A), Ala237(A), Leu347(A), Ile218(A), and other surrounding residues. The 3D structure shows curcumin (cyan) positioned within the IRAK1 binding pocket, maximizing interactions with key amino acids.

The docking analysis of curcumin with IRAK1 revealed a strong binding affinity, with a docking score of −9.05 kcal/mol and an RMSD of 2.00 Å (Table 4). The 2D interaction diagram (Figure 11, left) shows that curcumin forms multiple hydrogen bonds with key residues in the IRAK1 active site, including Ser295(A) and Asp358(A). These polar interactions are complemented by an extensive network of hydrophobic contacts with residues such as Tyr288(A), Val272(A), Leu291(A), Ala237(A), Leu347(A), Ile218(A), and others, as indicated by the red arcs.

The 3D visualization (Figure 11, right) further confirms that curcumin is well accommodated in the binding pocket of IRAK1. The ligand (depicted in cyan) is oriented to maximize both hydrogen bonding and hydrophobic interactions, with its aromatic rings and polar groups positioned in close proximity to critical residues, including LEU 291, VAL 272, LEU 347, SER 295, ASP 298, SER 344, ASP 358, LYS 239, TYR 288, and PHE 223.

Overall, these results suggest that curcumin can effectively interact with IRAK1 through a combination of hydrogen bonds and hydrophobic contacts, supporting its potential as a potent natural inhibitor of IRAK1 in inflammatory processes such as sepsis. These findings highlight IRAK1 as a promising molecular target for curcumin in the context of sepsis, supporting the rationale for further investigation of curcumin–IRAK1 interactions.

**Figure 12:**
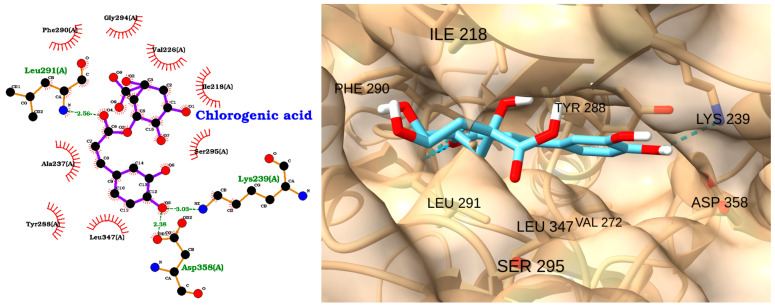
Two-dimensional interaction diagram (**left**) and three-dimensional binding pose (**right**) of chlorogenic acid docked to the IRAK1 active site. The 2D diagram highlights hydrogen bonds (dashed green lines) between chlorogenic acid and residues Leu291(A), Lys239(A), and Asp358(A), as well as hydrophobic interactions (red arcs) with Phe290(A), Ala237(A), Tyr288(A), Leu347(A), Gly294(A), and Val272. The 3D structure shows chlorogenic acid (cyan) positioned within the IRAK1 binding pocket, forming key contacts with the labeled residues.

Chlorogenic acid, a polyphenolic compound abundant in dietary sources and herbal extracts such as *Lonicerae flos*, has demonstrated significant anti-inflammatory and organ-protective effects in preclinical models of sepsis and related inflammatory disorders [85]. In rodent models of sepsis and endotoxemia, the administration of chlorogenic acid markedly improved survival rates, reduced multiorgan injury, and attenuated acute liver damage [86,87]. Collectively, these findings support the rationale for investigating the molecular interactions between chlorogenic acid and IRAK kinases, including IRAK1. The docking analysis of chlorogenic acid with IRAK1 indicates a moderate binding affinity, with a docking score of −7.14 kcal/mol and an RMSD of 2.13 Å (Table 4). The 2D interaction diagram (Figure 12, left) reveals that chlorogenic acid forms several key hydrogen bonds with residues in the IRAK1 active site, including Leu291(A), Lys239(A), and Asp358(A). These polar interactions are complemented by a network of hydrophobic contacts with residues such as Phe290(A), Ala237(A), Tyr288(A), Leu347(A), Gly294(A), and Val272, which further stabilize the ligand within the binding pocket.

The 3D visualization (Figure 12, right) confirms that chlorogenic acid is well accommodated in the IRAK1 binding pocket. The ligand (shown in cyan) is oriented to maximize both hydrogen bonding and hydrophobic interactions, with its aromatic rings and polar groups positioned in close proximity to the key interacting residues, including ILE 218, PHE 290, TYR 288, LEU 291, LEU 347, VAL 272, SER 295, LYS 239, and ASP 358.

Overall, these results suggest that chlorogenic acid could interact effectively with IRAK1 through a combination of hydrogen bonds and hydrophobic contacts, supporting its potential as a natural modulator of IRAK1 activity in inflammatory processes.

### 3.4. Docking of NAOs with Caspase-3

Sepsis-induced apoptosis and oxidant/antioxidant status is a well-recognized phenomenon, contributing significantly to immune cell depletion and organ failure [3]. In sepsis, excessive or inappropriate apoptosis of immune cells and parenchymal cells contributes to immune dysfunction, organ damage, and ultimately, increased mortality In in vivo studies have demonstrated that reducing inhibition of caspase-3 can improve septic lung injury in mice models [88]. Therefore, modulating Caspase-3 activity could be a potential therapeutic strategy for mitigating the detrimental effects of sepsis [89]. Previous studies have utilized molecular docking approaches to identify natural compounds capable of inhibiting caspase-3, with the aim of discovering small molecules that can modulate caspase activity and thereby contribute to the treatment of apoptosis-related and inflammatory diseases [90]. Despite the therapeutic promise of caspase-3 inhibitors for treating apoptosis-related and inflammatory diseases, the clinical development of synthetic caspase inhibitors has been hampered by significant challenges, including organ toxicity, poor metabolic stability, and limited membrane permeability [91]. These setbacks underscore the urgent need to identify alternative caspase-3 inhibitors with improved safety and tolerability. Focusing on NAOs with well-established clinical safety could accelerate the development of effective and safe caspase-3 inhibitors for the treatment of apoptosis-driven and inflammatory conditions, overcoming the limitations associated with synthetic agents.

Among the natural compounds, curcumin and EGCG exhibited the strongest predicted binding affinities, with docking scores of −6.23 and −6.22 kcal/mol, respectively (Table 5). Curcumin, however, showed a higher RMSD (2.71 Å), suggesting a less stable binding pose compared to EGCG (RMSD: 1.79 Å). Resveratrol demonstrated a moderate binding affinity (−5.49 kcal/mol) but had the lowest RMSD (0.16 Å), indicating a highly stable and reproducible docking pose. Quercetin and chlorogenic acid showed moderate binding affinities and pose stabilities. The reference inhibitor CHEMBL456799 achieved the best docking score (−7.39 kcal/mol), serving as a benchmark for strong binding, though its RMSD (2.91 Å) was higher than most natural compounds (Table 5). The negative compound CHEMBL1242700 had a moderate docking score (−5.59 kcal/mol) and the highest RMSD (3.99 Å), indicating less stable binding (Table 5).

**Figure 13:**
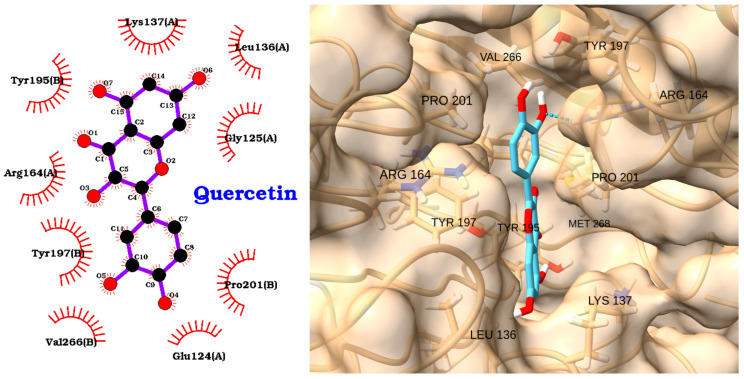
Two-dimensional interaction diagram (**left**) and three-dimensional binding pose (**right**) of quercetin docked to the caspase-3 active site. The 2D diagram highlights hydrophobic interactions (red arcs) between quercetin and residues Lys137(A), Leu136(A), Gly125(A), Glu124(A), Tyr195(B), Arg164(A), Tyr197(B), Pro201(B), and Val266(B). The 3D structure shows quercetin (cyan) positioned within the caspase-3 binding pocket, forming key contacts with the labeled residues.

The docking analysis of quercetin with caspase-3 revealed a moderate binding affinity, with a docking score of −5.66 kcal/mol and an RMSD of 1.35 Å (Table 5). The 2D interaction diagram (Figure 13, left) shows that quercetin is stabilized within the caspase-3 binding pocket primarily through multiple hydrophobic interactions with key residues, including Lys137(A), Leu136(A), Gly125(A), Glu124(A), Tyr195(B), Arg164(A), Tyr197(B), Pro201(B), and Val266(B). These interactions help anchor the quercetin molecule in a favorable orientation within the active site.

The 3D visualization (Figure 13, right) further confirms that quercetin fits well into the caspase-3 binding pocket, with its planar aromatic structure aligned along the groove formed by residues such as VAL266, PRO201, TYR197, ARG164, LEU136, and LYS137. The low RMSD value indicates a stable docking pose, supporting the reproducibility of the predicted binding mode. Overall, these results suggest that quercetin can interact effectively with caspase-3, primarily via hydrophobic contacts, supporting its potential as a natural modulator of caspase-3 activity in apoptosis-related and inflammatory conditions.

Several studies provide experimental and computational support for quercetin as a modulator of caspase-3 activity [92,93,94]. Experimental research further confirms that quercetin induces apoptosis in various cancer and endothelial cell lines through the activation of the caspase-dependent pathway, including the upregulation and cleavage of caspase-3, as well as PARP, even in the presence of caspase-8 and -9 inhibitors [95,96]. In addition, quercetin has been shown to alleviate apoptosis and inflammation in vascular and other cell types by downregulating caspase-3 expression and activity, and by modulating upstream regulators such as NF-κB and AP-1 [92,97,98]. These findings collectively support the relevance of quercetin as a natural compound capable of interacting with and modulating caspase-3, aligning with the results of the present docking study.

**Figure 14:**
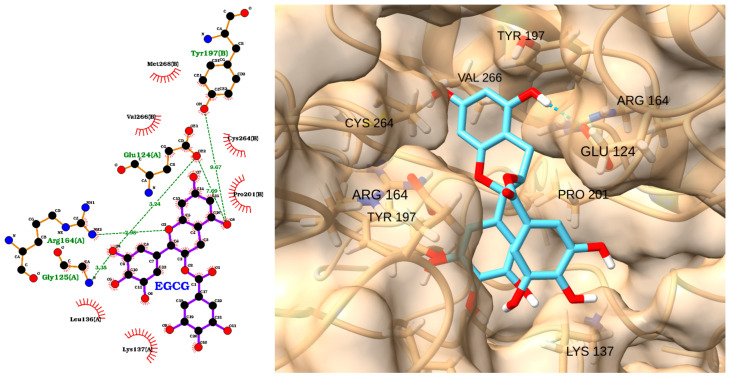
Two-dimensional interaction diagram (**left**) and three-dimensional binding pose (**right**) of EGCG docked to the caspase-3 active site. The 2D diagram highlights hydrogen bonds (dashed green lines) between EGCG and residues Glu124(A), Arg164(A), Gly125(A), and Tyr197(B), as well as hydrophobic interactions (red arcs) with Val266(B), Pro201(B), Tyr195(B), Leu136(A), and Lys137(A). The 3D structure shows EGCG (cyan) positioned within the caspase-3 binding pocket, forming key contacts with the labeled residues.

EGCG, a major polyphenol in green tea, has been widely studied for its anti-apoptotic and anti-inflammatory properties. Several studies have shown that EGCG can modulate caspase-3 activity both in vitro and in vivo [99,100]. For example, EGCG has been reported to inhibit caspase-3 activation and apoptosis in neuronal and endothelial cells exposed to oxidative stress or inflammatory stimuli, thereby conferring neuroprotective and cytoprotective effects [101]. The docking analysis of EGCG (epigallocatechin gallate) with caspase-3 revealed a strong binding affinity, with a docking score of −6.22 kcal/mol and an RMSD of 1.79 Å (Table 5). The 2D interaction diagram (Figure 14, left) shows that EGCG forms multiple hydrogen bonds with key residues in the caspase-3 active site, including Glu124(A), Arg164(A), Gly125(A), and Tyr197(B). These polar interactions are complemented by extensive hydrophobic contacts with residues such as Val266(B), Pro201(B), Tyr195(B), Leu136(A), and Lys137(A). The 3D visualization (Figure 14, right) confirms that EGCG fits snugly within the caspase-3 binding pocket, with its aromatic rings and hydroxyl groups optimally oriented to maximize both hydrogen bonding and hydrophobic interactions. Overall, these results suggest that EGCG could effectively interact with caspase-3, supporting its potential as a natural modulator of caspase-3 activity in apoptosis-related and inflammatory conditions.

Resveratrol, a polyphenolic compound found in grapes and red wine, has been widely studied in sepsis for its anti-apoptotic and anti-inflammatory properties [102]. Resveratrol enhances the expression of endogenous Klotho, thereby exerting antiapoptotic effects that protect the kidneys of mice from sepsis-induced acute kidney injury [103]. Resveratrol inhibited all three proteolytic activities of the proteasome with varying IC_50_ values, showing a particular preference for LMP7. This inhibition led to the accumulation of polyubiquitinated proteins and phosphorylated IκBα in CD14^+^ monocytes and supports the use of resveratrol as a potential natural therapeutic drug for treating the early stages of sepsis [104]. Although preclinical studies have demonstrated that resveratrol can modulate inflammatory and oxidative pathways and protect against organ damage in sepsis models, the precise mechanisms underlying its beneficial effects remain to be fully elucidated. Therefore, further research—particularly well-designed human studies—is essential to clarify how resveratrol functions in sepsis and to validate its therapeutic potential in clinical settings [105]. These findings support our molecular docking studies between resveratrol and the caspase-3 active site.

**Figure 15:**
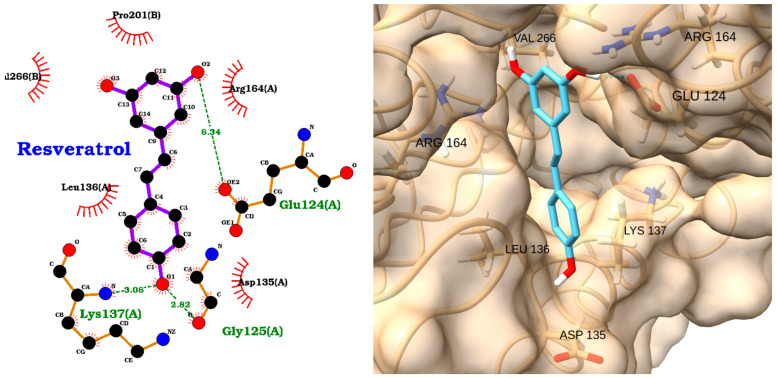
Two-dimensional interaction diagram (**left**) and three-dimensional binding pose (**right**) of resveratrol docked to the caspase-3 active site. The 2D diagram highlights hydrogen bonds (dashed green lines) between resveratrol and residues Glu124(A), Lys137(A), and Gly125(A), as well as hydrophobic interactions (red arcs) with Val266(B), Leu136(A), Pro201(B), Arg164(A), and Asp135(A). The 3D structure shows resveratrol (cyan) positioned within the caspase-3 binding pocket, forming key contacts with the labeled residues.

The docking analysis of resveratrol with caspase-3 revealed a moderate binding affinity, with a docking score of −5.49 kcal/mol and an exceptionally low RMSD of 0.16 Å (Table 5), indicating a highly stable and reproducible binding pose. The 2D interaction diagram (Figure 15, left) shows that resveratrol forms three key hydrogen bonds with the active site residues: Glu124(A) (2.34 Å), Lys137(A) (3.06 Å), and Gly125(A) (2.82 Å). Additionally, resveratrol is stabilized by multiple hydrophobic interactions with residues such as Val266(B), Leu136(A), Pro201(B), Arg164(A), and Asp135(A).

The 3D visualization (Figure 15, right) further confirms that resveratrol is well accommodated within the caspase-3 binding pocket, with its planar aromatic structure fitting snugly among the key interacting residues, including VAL 266, ARG 164, GLU 124, LYS 137, LEU 136, and ASP 135. The combination of hydrogen bonding and hydrophobic contacts supports the predicted stable and favorable binding mode.

Overall, these results suggest that resveratrol could effectively interact with caspase-3, supporting its potential as a natural modulator of caspase-3 activity and highlighting its promise for therapeutic applications in apoptosis-related and inflammatory diseases.

Curcumin, the principal polyphenol in turmeric, has been extensively studied for its anti-inflammatory and anti-apoptotic effects. Multiple experimental studies have demonstrated that curcumin can modulate caspase-3 activity, either by inhibiting excessive apoptosis in models of neurodegeneration and tissue injury or by promoting apoptosis in cancer cells. For example, curcumin has been shown to suppress caspase-3 activation and neuronal apoptosis in models of cerebral ischemia and to induce caspase-3-mediated apoptosis in melanoma and carcinoma cells [106,107]. Significant advances have been made in understanding the effects of curcumin in sepsis using rodent and in vitro cellular models. However, clinical evidence for curcumin’s efficacy in the management of sepsis remains limited and inconclusive, with only a handful of trials—often using nano-formulations—showing modest improvements in inflammatory biomarkers and clinical scores in critically ill patients [108]. This gap between promising preclinical findings and limited clinical translation highlights the need for deeper mechanistic insights into how curcumin interacts with molecular targets relevant to sepsis pathophysiology. Computational analyses can help identify specific binding sites, affinities, and modes of action, providing a molecular rationale for curcumin’s observed biological effects and guiding the design of more targeted experimental and clinical studies.

**Figure 16:**
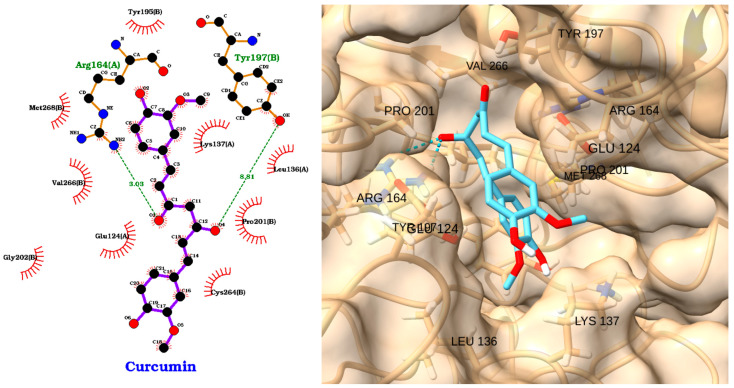
Two-dimensional interaction diagram (**left**) and three-dimensional binding pose (**right**) of curcumin docked to the caspase-3 active site. The 2D diagram highlights hydrogen bonds (dashed green lines) between curcumin and residues Arg164(A) and Tyr197(B), as well as hydrophobic interactions (red arcs) with Val266(B), Met268(B), Pro201(B), Leu136(A), Tyr195(B), Glu124(A), and Lys137(A). The 3D structure shows curcumin (cyan) positioned within the caspase-3 binding pocket, forming key contacts with the labeled residues.

The docking analysis of curcumin with caspase-3 revealed a strong binding affinity, with a docking score of −6.23 kcal/mol and an RMSD of 2.71 Å (Table 5). The 2D interaction diagram (Figure 16, left) shows that curcumin forms key hydrogen bonds with Arg164(A) and Tyr197(B), which help anchor the molecule within the caspase-3 active site. In addition to these polar interactions, curcumin is stabilized by a network of hydrophobic contacts with residues such as Val266(B), Met268(B), Pro201(B), Leu136(A), Tyr195(B), Glu124(A), and Lys137(A).

The 3D visualization (Figure 16, right) further confirms that curcumin fits well within the caspase-3 binding pocket, with its planar structure and polar groups optimally oriented to engage both hydrogen bonding and hydrophobic interactions with the surrounding residues, including VAL266, PRO201, ARG164, GLU124, LEU136, LYS137, and TYR197. Molecular docking and simulation studies further support curcumin’s ability to bind directly to the caspase-3 active site, forming stable hydrogen bonds and hydrophobic interactions with key catalytic residues [109]. These findings are consistent with the present docking results, highlighting curcumin as a promising natural modulator of caspase-3 activity with potential therapeutic applications in apoptosis-related and inflammatory diseases.

**Figure 17:**
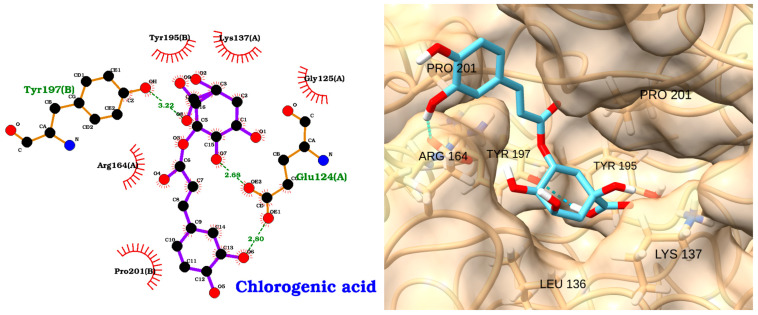
Two-dimensional interaction diagram (**left**) and three-dimensional binding pose (**right**) of chlorogenic acid docked to the caspase-3 active site. The 2D diagram highlights hydrogen bonds (dashed green lines) between chlorogenic acid and residues Tyr197(B), Glu124(A), and Pro201(B), as well as hydrophobic interactions (red arcs) with Tyr195(B), Tyr137(A), Gly125(A), Arg164(A), and Pro201(B). The 3D structure shows chlorogenic acid (cyan) positioned within the caspase-3 binding pocket, forming key contacts with the labeled residues.

Chlorogenic acid, a polyphenolic compound found in coffee and many plant-based foods, has been reported to inhibit caspase-3 activation and reduce apoptosis in models of hepatic and neuronal injury [110,111,112]. Molecular docking and simulation studies further support its ability to bind directly to the caspase-3 active site, forming stable hydrogen bonds and hydrophobic interactions with key residues [113]. The docking analysis of chlorogenic acid with caspase-3 revealed a moderate binding affinity, with a docking score of −5.28 kcal/mol and an RMSD of 1.93 Å (Table 5). The 2D interaction diagram (Figure 17, left) shows that chlorogenic acid forms several key hydrogen bonds with active site residues, including Tyr197(B) (3.22 Å), Glu124(A) (2.86 Å), and Pro201(B) (2.50 Å). These polar interactions are complemented by a network of hydrophobic contacts with residues such as Tyr195(B), Tyr137(A), Gly125(A), Arg164(A), and Pro201(B), which further stabilize the ligand within the caspase-3 binding pocket.

The 3D visualization (Figure 17, right) confirms that chlorogenic acid fits well within the caspase-3 binding pocket, with its aromatic and polar groups optimally oriented to engage both hydrogen bonding and hydrophobic interactions with surrounding residues, including PRO201, ARG164, TYR197, TYR195, LEU136, and LYS137.

A recent study by Kimsa-Dudek et al. (2022) demonstrated that both chlorogenic acid and caffeic acid exhibit significant pro-apoptotic activity in C32 melanoma cells exposed to a static magnetic field, as shown by increased expression of caspase-3 and caspase-9 and downregulation of anti-apoptotic genes such as *Bcl2* and *BclXl* [114]. Molecular docking analyses revealed that both phenolic acids have a strong molecular affinity for proteins associated with apoptosis pathways, supporting their mechanistic role as pro-apoptotic modulators [115]. These findings are consistent with the present docking results, highlighting chlorogenic acid as a promising natural modulator of caspase-3 activity with potential therapeutic applications in apoptosis-related and inflammatory diseases.

## 4. Conclusions

This study systematically evaluated the molecular interactions of five selected natural antioxidant compounds (quercetin, EGCG, resveratrol, curcumin, and chlorogenic acid) with three key proteins implicated in inflammatory and apoptotic pathways-TLR-4, IRAK1, and caspase-3-using molecular docking analyses. The docking scores and binding poses revealed that all five NAOs possess the potential to interact favorably with these receptors, albeit with varying affinities and stabilities.

Among the tested compounds, curcumin and EGCG consistently demonstrated strong binding affinities across multiple targets, with docking scores and interaction profiles that often approached or exceeded those of reference inhibitors, particularly for IRAK1 and caspase-3. Resveratrol showed moderate binding affinities but exhibited highly stable docking poses, especially with caspase-3, suggesting a reproducible and potentially effective interaction. Quercetin and chlorogenic acid also exhibited moderate affinities and stable binding modes, supporting their potential as multi-target modulators.

The detailed interaction analyses revealed that these NAOs engage in both hydrogen bonding and hydrophobic contact with critical residues within the binding pockets of TLR-4, IRAK1, and caspase-3. Such interactions are consistent with the known anti-inflammatory and anti-apoptotic mechanisms of these compounds reported in experimental studies. Notably, the docking results for caspase-3 align with literature evidence supporting the ability of quercetin, EGCG, resveratrol, curcumin, and chlorogenic acid to modulate apoptosis through caspase-dependent pathways.

Taken together, these computational findings suggest that NAOs may exert therapeutic effects in sepsis by targeting multiple proteins involved in its pathogenesis and may guide the rational design of novel therapeutic strategies targeting TLR-4, IRAK1, and caspase-3 in inflammatory and apoptosis-related diseases. However, it is important to acknowledge that this study is based solely on computational models, and experimental validation is essential to confirm these interactions, assess actual binding affinities, and fully elucidate the therapeutic potential of NAOs in sepsis. Specifically, future studies should focus on validating binding and activity in cell-based and in vivo models, as well as investigating dose–response relationships and potential synergistic effects between different NAOs or with conventional therapies.

## Figures and Tables

**Table 1:** Summary of selected receptors for sepsis study.

Receptor	Uniprot ID	Role in Sepsis	Mechanism Represented
TLR4	O00206	Innate immunity	Recognition of LPS, initiating immune response
IRAK1	P51617	Inflammation	Involved in TLR and IL-1R signaling pathways
Caspase-3	P42574	Apoptosis	Key enzyme in programmed cell death

**Table 2:** Natural antioxidants (NAOs) and their potential roles in sepsis-related diseases. The PubChem accession numbers (CID) and clinical trial IDs are included.

NAO	CID	Potential Role in Sepsis-Related Diseases	Clinical Trial
Quercetin	5280343	Chronic obstructive pulmonary disease	NCT01708278
EGCG	65064	Prophylaxis of influenza infection	NCT01008020
Resveratrol	445154	Inflammation and oxidative stress in chronic kidney disease	NCT02433925
Curcumin	969516	Modulating gut microbiota, reducing endotoxemia	NCT03329781
Chlorogenic acid	1794427	Renal insufficiency	NCT02524938

**Table 3:** Docking scores (in kcal/mol) and root-mean-square deviation (RMSD, in Å) for natural antioxidants (NAOs) and control compounds docked to TLR-4. More negative docking scores indicate a stronger predicted binding affinity, while lower RMSD values reflect greater stability and reproducibility of the ligand-binding pose.

Ligand	Score	RMSD
Quercetin	−5.20	2.279
EGCG	−5.15	0.3575
Resveratrol	−4.60	2.122
Curcumin	−4.46	1.455
Chlorogenic_acid	−3.76	2.655714
CHEMBL5174883 (Positive)	−4.22	1.552
Carvedilol (Negative)	−3.41	2.81

**Table 4:** Docking scores (in kcal/mol) and root-mean-square deviation (RMSD), in Å, for natural antioxidants (NAOs) and IRAK1, using the DL1 ligand co-crystallized in the PDB structure as a positive control. More negative docking scores indicate stronger predicted binding affinity, while lower RMSD values reflect greater stability and reproducibility of the ligand binding pose.

Ligand	Score	RMSD
Quercetin	−8.18	0.269
EGCG	−8.86	1.8475
Resveratrol	−6.72	0.379
Curcumin	−9.05	2
Chlorogenic_acid	−7.14	2.1275
DL1 ligand in PDB	−9.30	0.792

**Table 5:** Docking scores (in kcal/mol) and root-mean-square deviation (RMSD, in Å) for natural antioxidants (NAOs) and Caspase-3, using the compounds CHEMBL456799 and CHEMBL1242700 as a positive and negative control, respectively. More negative docking scores indicate stronger predicted binding affinity, while lower RMSD values reflect greater stability and reproducibility of the ligand binding pose.

Ligand	Score	RMSD
Quercetin	−5.66	1.35125
EGCG	−6.22	1.786667
Resveratrol	−5.49	0.156
Curcumin	−6.23	2.71
Chlorogenic_acid	−5.28	1.926667
CHEMBL456799 (positive)	−7.39	2.908
CHEMBL1242700 (negative)	−5.59	3.9875

## Data Availability

The original contributions presented in this study are included in the article. Further inquiries can be directed to the corresponding author(s).
